# Genome-Wide Association Uncovered SbERF60 Positively Regulates Mesocotyl Length in Sorghum

**DOI:** 10.3390/plants15132000

**Published:** 2026-06-28

**Authors:** Lan Ju, Xiaoqiang Cheng, Yao Wang, Ruizhen Liu, Haisheng Yan, Yubin Wang, Jianqiang Chu, Fangfang Fan, Xin Lv, Hao Niu, Junai Ping

**Affiliations:** 1 Hou Ji Laboratory in Shanxi Province, Sorghum Research Institute of Shanxi Agricultural University, Jinzhong 030600, China; 2 Key Laboratory of Minor Crop Germplasm Innovation and Molecular Breeding, Ministry of Agriculture and Rural Affairs, Taiyuan 030032, China; 3 College of Agriculture, Shanxi Agricultural University, Jinzhong 030801, Chinairz020316@163.com (R.L.)

**Keywords:** sorghum, mesocotyl length, deep-seeding emergence, genome-wide association studies, SbERF60

## Abstract

Mesocotyl length is a key factor affecting deep-seedling emergence and consequently influences grain yield in sorghum. However, at present, limited genes regulating mesocotyl elongation have been identified and the underlying molecular mechanisms remain unclear in sorghum. Here, we combined a genome-wide association study (GWAS) on 232 diverse sorghum accessions with transcriptomic analysis to identify key genes controlling mesocotyl length. We identified SbERF60, an AP2/ERF transcription factor, as a strong candidate based on its functional annotation and differential expression patterns in sorghum varieties with contrasting mesocotyl lengths and its increased transcript levels during mesocotyl development. It was discovered that natural variation in its promoter enhances *SbERF60* expression by increasing promoter activity, thereby leading to longer mesocotyl length. Functional validation confirmed that *SbERF60* promotes mesocotyl elongation and significantly improves deep-seeding emergence in rice. These findings provide valuable genetic insights and identify a key target for molecular breeding strategies to improve sorghum deep-seedling emergence.

## 1. Introduction

Sorghum [*Sorghum bicolor* (L.) Moench], as an essential cereal crop, is highly valued for its exceptional drought tolerance. In order to cope with drought stress, deep sowing is really necessary [1]. However, this could adversely affect sorghum deep-seedling emergence, consequently causing a notable decrease in production [2]. In recent years, with the widespread adoption of mechanized farming and single-seed precision sowing, this issue has become more serious. Despite its agronomic importance, research on sorghum emergence rates remains very limited.

Mesocotyl serves as the essential structure that determines seedling emergence under deep soil [3,4,5,6,7]. Therefore, developing varieties with long mesocotyls has become a major objective in current sorghum breeding programs. And identifying key genes controlling mesocotyl length (ML) is essential for the molecular breeding of sorghum varieties with long mesocotyls.

Genome-wide association studies (GWAS) offer a powerful method for detecting links between genetic variations across the genome and observable traits in populations with genetic diversity [8,9,10]. In recent years, the application and development of GWAS in crops have provided opportunities for identifying key genes relating to mesocotyl traits. To date, several quantitative trait loci (QTLs) regulating mesocotyl length have been identified through GWAS in rice and maize, establishing a basis for developing cultivars with enhanced tolerance to deep sowing [11,12,13]. Subsequently, two key genes controlling mesocotyl length were subsequently identified through GWAS in rice. The *OsML1*, a mitochondrial transcription termination factor (mTERF) gene, contributes to mesocotyl length by promoting cell elongation through H_2_O_2_ homeostasis [6]. *OsGSK2*, a conserved glycogen synthase kinase 3 (GSK3)-like kinase, promotes cell elongation by coordinately regulating strigolactone and brassinosteroid signaling pathways, ultimately promoting mesocotyl length [14].

In addition to QTLs, multiple genes contributing to mesocotyl length variation have been identified in rice [5,15]. The suppression of *OsGY1* leads to a decrease in jasmonic acid (JA) levels, leading to the promotion of mesocotyl elongation [16]. *OsPAO5* restricts cell expansion by inducing cell wall hardening, thereby inhibiting mesocotyl elongation [17]. *OsSMAX1* promotes mesocotyl elongation by negatively regulating the Strigolactone (SL) biosynthesis pathway [18]. *OsEXP4* promotes mesocotyl elongation, and its overexpression increases mesocotyl length by approximately 97% [19].

The APETALA2/ethylene responsive factor (AP2/ERF) family comprises plant-specific transcription factors that contain a minimum of one highly conserved DNA-binding domain, typically spanning 60 to 70 amino acids in length. They play diverse roles in growth, hormone pathways, and stress adaptation [20,21]. ERF transcription factors have been widely implicated in regulating hypocotyl and mesocotyl elongation in plants [22,23,24].

In sorghum, a total of 158 ERF transcription factors has been identified within the genome, comprising 106 members from the ERF subfamily and 52 from the C-repeat binding factor/dehydration-responsive element binding (CBF/DREB) subfamily [21]. Nevertheless, the involvement of ERF transcription factors in controlling mesocotyl length in sorghum, along with the associated molecular mechanisms, is still unclear.

In this study, we identified SbERF60, an AP2/ERF transcription factor, as a key gene regulating mesocotyl length through a genome-wide association study of 232 diverse sorghum accessions combined with transcriptomic analysis. We further demonstrated that the natural variation in its promoter enhances *SbERF60* expression by increasing promoter activity, leading to longer mesocotyls. Through the transgenic rice lines overexpressing *SbERF60* we generated, we confirmed that *SbERF60* promotes mesocotyl elongation and significantly improves deep-seedling emergence in rice. Our work elucidates a molecular mechanism underlying mesocotyl development and establishes *SbERF60* as a valuable genetic resource for molecular breeding programs aimed at enhancing seedling establishment in sorghum.

## 2. Results

### 2.1. GWAS of Mesocotyl Phenotype in Sorghum

**Figure 1:**
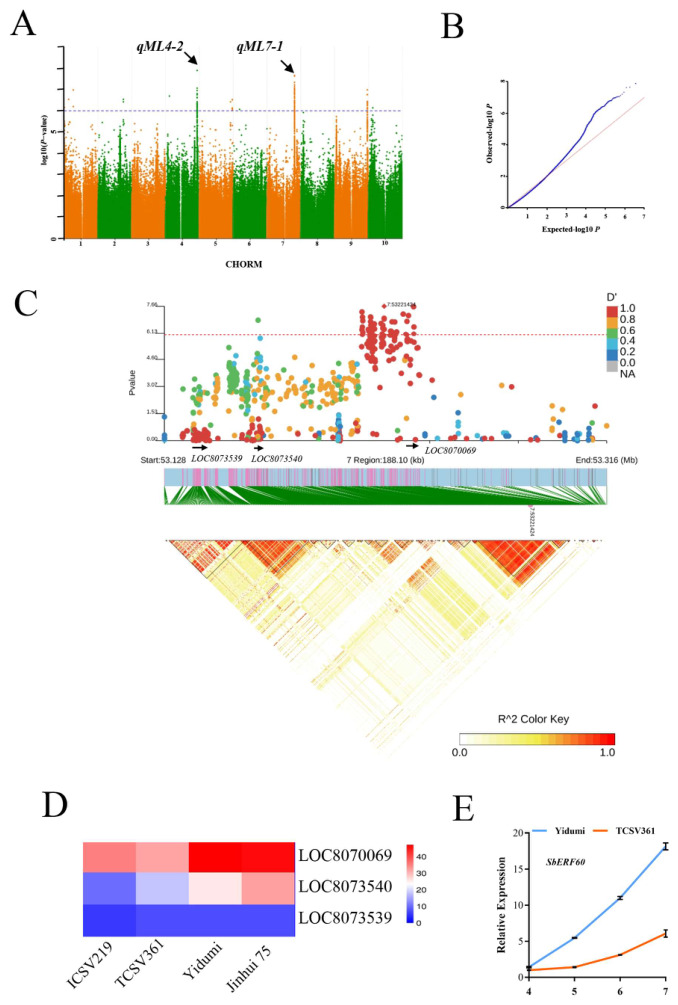
GWAS of mesocotyl length. (**A**) Manhattan plots and (**B**) Quantile-quantile plots of GWAS for ML using a linear mixed model (LMM), the dashed horizontal lines indicate the genome-wide significance threshold (**C**) Linkage disequilibrium (LD) block analysis of chr7. Dashed lines represent the significance threshold for linkage disequilibrium (LD) decay. The peak SNP is located at position 52,331,424 on chromosome 7 (7:52331424). (**D**) Heat map showing expression changes in candidate genes in contrasting genotypes by length using RNA-seq. (**E**) Expression changes in *SbERF60* in mesocotyls at different developmental days.

To further identify potential genes involved in the regulation of sorghum mesocotyl elongation, we conducted a genome-wide association study (GWAS) focusing on ML using a globally diverse panel of 232 sorghum germplasm accessions [25]. The frequency distribution plot revealed that the phenotypic values for ML exhibited a near-normal distribution, confirming their appropriateness for GWAS (Figure S1). The mesocotyl length in sorghum exhibited significant phenotypic variation, ranging from 1.9 cm to 16.5 cm (Figure S2). A total of 3,641,771 SNPs were analyzed for genome-wide associations using GAPIT version 3 using a mixed linear model (LMM). To control for multiple testing, raw *p* values were subjected to Bonferroni correction, yielding a genome-wide significance threshold of *p* < 1.192 × 10^−6^ (0.05/3,641,771). Under this threshold, 10 SNPs distributed across chromosomes 1, 2, 4, 5, 7, 9, and 10 were found to be significantly associated with mesocotyl elongation. Strong linkage disequilibrium (LD) was observed among these SNPs, and a total of 60 candidate genes were identified within their flanking regions. The LD decay rate was 34 kb when it decayed to 20%, indicating that 34,000 bp before and after the threshold point was selected as the candidate region significantly associated with the trait. Based on the above results, we focused on two QTLs with higher −log10(*p*) values, located on chromosome 4 (the second interval) and chromosome 7, named *qML4-2* and *qML7-1*, respectively (Figure 1A,B). Further analysis of the *qML7-1* revealed that this region harbors three high-confidence candidate genes (Figure 1C, Table S2).

### 2.2. Candidate Gene Identification by Integrating GWAS and Transcriptome Data

In a previous study, we conducted transcriptome analysis for four sorghum accessions with contrasting mesocotyl length phenomena [26]. Additionally, we examined the transcriptome data to assess the expression levels of the three candidate genes located on chromosome 7. Among these, we observed that *LOC8073540* was consistently upregulated in long-mesocotyl sorghum varieties relative to those with short mesocotyls. There was no significant difference in the expression levels of *LOC8073539* and *LOC8070069* in the extremely long material compared with those in the extremely short material (Figure 1D and Figure S3). To further investigate the *SbERF60* expression, we analyzed its expression changes at different developmental stages of the mesocotyl using two sorghum accessions, Yidumi (with an extremely long mesocotyl) and TCSV361 (with an extremely short mesocotyl). The results showed that the expression level of *SbERF60* was significantly higher in Yidumi than in TCSV361 at all time points during mesocotyl development. Additionally, the expression of this gene exhibited an increasing trend along with the growth and development of the mesocotyl in both accessions (Figure 1E). Previous research has suggested that five ERF genes could play a crucial role in modulating ML in sorghum [26]. Supporting this, an ERF transcription factor (*LOC_Os09g20350*) in rice was identified as a high-confidence regulator of mesocotyl elongation via GWAS and QTL analysis [15]. Based on these findings, we propose *LOC8073540* (*SbERF60*) as a promising candidate gene influencing ML in sorghum.

### 2.3. Spatial Expression of SbERF60 and Protein Subcellular Localization

**Figure 2:**
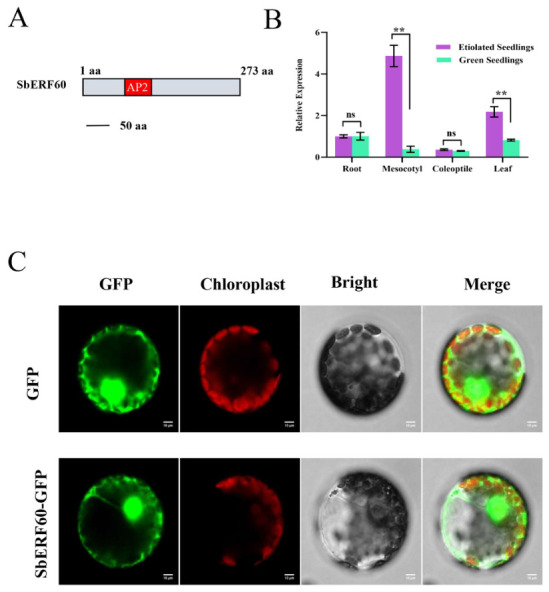
SbERF60 expression analysis. (**A**) Gene structure of *SbERF60*. (**B**) Relative expression level of *SbERF60* in different tissues of Yidumi. Etiolated seedlings and green seedlings represent seedlings cultured under regular water conditions in darkness and in 16 h light/8 h dark, respectively. (**C**) Subcellular localization of SbERF60 in *Nicotiana benthamiana* protoplasts, showing the merged green fluorescence of GFP fusion protein. The 35S-GFP is used as a control. Asterisks indicate a significant difference according to Student’s *t* test (** *p* < 0.01; ns = non-significant).

To elucidate the function of SbERF60, we cloned this gene and analyzed its structural features. Sequence analysis from the NCBI database revealed that SbERF60 contains no intron and encodes a protein of 273 amino acid residues featuring a conserved AP2 domain (Figure 2A). We further investigated its expression pattern by quantitative real-time PCR (qPCR). *SbERF60* was ubiquitously expressed across all organs examined. Notably, in etiolated seedlings, its transcript level was significantly higher in the mesocotyl compared with other tissues. Additionally, expression levels in green seedlings were lower than those in etiolated seedlings (Figure 2B).

To examine the subcellular distribution of SbERF60, a transient expression experiment was performed using *Nicotiana benthamiana* (*N. benthamiana*) protoplasts. The plasmid *pBWA(V)H2STMVΩ-SbERF60* was employed, where the coding sequence of SbERF60 was fused to Green Fluorescent Protein (GFP), generating an SbERF60-GFP protein. The results suggest that the fluorescence signal of GFP was observed in both the nuclei and cytoplasm, confirming that SbERF60 protein is mainly located in the nucleus and cytoplasm (Figure 2C).

### 2.4. Haplotype Analysis of the SbERF60 Promoter

**Figure 3:**
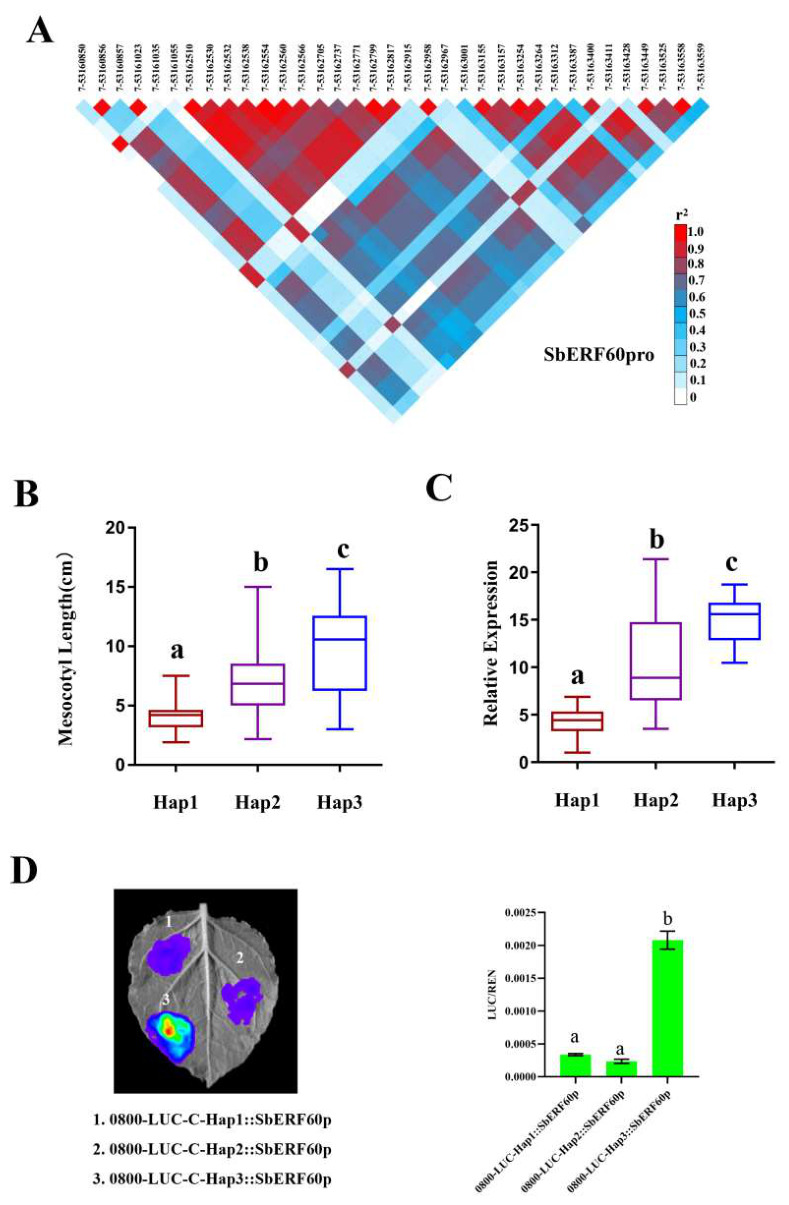
Natural variation in the *SbERF60* promoter was responsible for SbERF60 expression regulation. (**A**) The linkage disequilibrium map of SNP sites in the *SbERF60* promoter. (**B**) Mesocotyl length and expression level in different HAPs of *SbERF60*. (**C**) Level of the *SbERF60* gene in two haplotypes of *SbERF60* accessions. (**D**) Identification of the activity of promoters in different HAPs of *SbERF60*. Different lowercase letters above the bars indicate significant differences at the 0.01 probability level. Statistical significance between groups via one-way ANOVA with Tukey’s HSD post hoc test (*p* < 0.05). All data are presented as mean ± SD, with *n* = 3 biological replicates per haplotype.

Based on resequencing data from 232 sorghum accessions, 35 single nucleotide polymorphisms (SNPs) were identified in the SbERF60 promoter region. Linkage disequilibrium (LD) analysis of these SNP sites is presented as follows, whereas no SNPs were detected in the coding sequence (CDS) of SbERF60. For haplotype inference, all 35 SNPs were used to define haplotype blocks, and haplotypes with a frequency of <5% were excluded from subsequent analysis. Based on the distinct allelic combinations of the remaining SNPs, all accessions were classified into three major haplotypes, designated Hap1, Hap2, and Hap3, comprising 26, 90, and 35 accessions, respectively (Figure 3B). Phenotypic comparison of mesocotyl length across three haplotypes showed that the mesocotyl length of Hap3 was significantly longer than Hap1 and Hap2, indicating that Hap3 represents the favorable allele for longer mesocotyls (Figure 3B). To investigate whether haplotype variation influences gene expression, we analyzed the expression levels of *SbERF60* using 18 representative accessions from each haplotype. The results showed that *SbERF60* expression was significantly lower in Hap1 than in Hap2 and Hap3.

To further examine whether sequence variations affect promoter activity, the *SbERF60* promoter sequences from the three haplotypes were cloned into the pGreenII-0800-LUC vector for luciferase assays. The luciferase reporter system showed that the luciferase activity of Hap3 was significantly higher than that of Hap1 and Hap2 (Figure 3D). Collectively, these results indicate that natural sequence variation in the *SbERF60* promoter influences *SbERF60* expression by modulating promoter activity.

### 2.5. Overexpression of SbERF60 Promotes Mesocotyl Elongation in Rice

**Figure 4:**
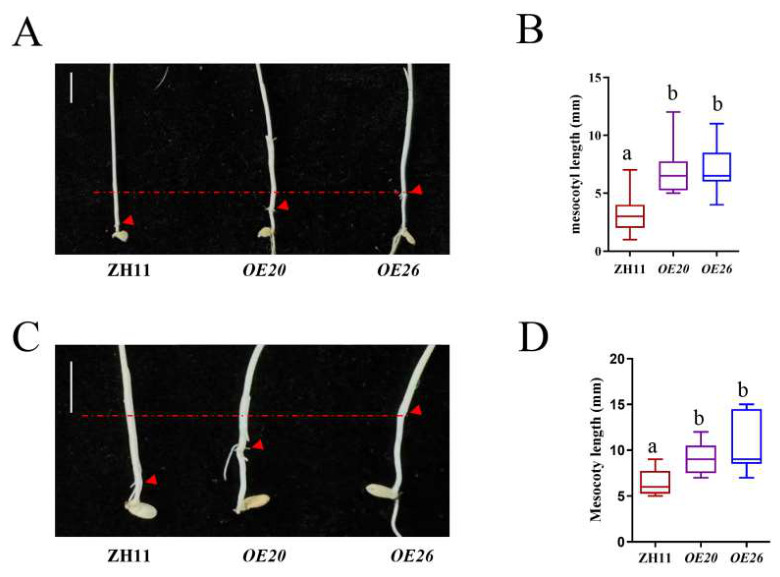
Overexpression of *SbERF60* promotes mesocotyl elongation in rice. (**A**,**B**) Phenotypic analysis of mesocotyl length in Zhonghua 11 and *SbERF60* overexpression rice lines under a sowing depth of 6 cm. (**C**,**D**) Phenotypic analysis of mesocotyl length in Zhonghua11 and *SbERF60* overexpression rice lines under a sowing depth of 8 cm. Arrows indicate the coleoptilar nodesDashed lines are added to **aid** visual comparison between different groups. Different lowercase letters above the bars indicate significant differences at the 0.01 probability level. Bar = 0.5 cm. Statistical significance between groups via one-way ANOVA with Tukey’s HSD post hoc test (*p* < 0.05). All data are presented as mean ± SD, with *n* = 3 biological replicates per haplotype and at least 8 seedlings per replicate.

In order to further clarify the functions of SbERF60 in regulating mesocotyl elongation, we constructed an overexpression vector *pUbi:SbERF60-3xFlag* and transformed it into Zhonghua 11 (ZH11), generating transgenic overexpressing lines (Figure S4). As shown in the figures, under a sowing depth of 6 cm, the mesocotyl length of the overexpression lines was significantly increased compared with wild-type plants (Figure 4A,B). When the sowing depth was increased to 8 cm, the mesocotyl length of the overexpression lines also showed a significant increase relative to the wild-type plants (Figure 4C,D). These results confirmed that *SbERF60* promotes mesocotyl elongation in rice.

### 2.6. SbERF60 Improves Deep Seeding Emergence

**Figure 5:**
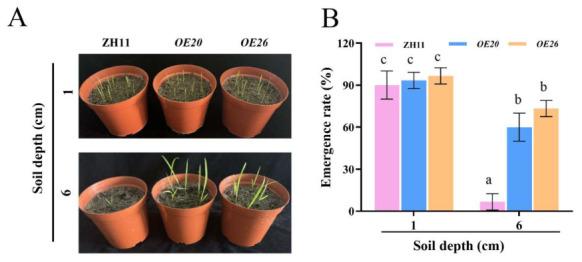
SbERF60 improves seedling emergence under deep seeding. (**A**) Effects of SbERF60 on seedling emergence. Images showing seeds of ZH11 at 7 days after sowing (DAS) and *OE20* and *OE26* lines at 13 DAS. (**B**) Emergence rates of the indicated lines measured at 13 DAS. Statistical significance between groups via one-way ANOVA with Tukey’s HSD post hoc test (*p* < 0.05). All data are presented as mean ± SD, with *n* = 3 biological replicates per haplotype and at least 15 seedlings per replicate.

To investigate whether the promoting effect of SbERF60 on mesocotyl elongation translates into improved deep-seedling emergence, we conducted deep-seeding emergence experiments using overexpression lines and their corresponding WT lines. The results indicated that at a sowing depth of 1 cm, both the overexpression lines and the wild-type plants emerged normally (Figure 5A,B). However, at a sowing depth of 6 cm, the overexpression lines showed significantly higher emergence rates than wild-type plants (Figure 5A,B). These results confirmed that SbERF60 enhances deep-sowing tolerance in rice.

## 3. Discussion

Sorghum is mainly cultivated in arid and semi-arid regions, and thus deep sowing plays a crucial role in coping with drought and ensuring seedling survival [1,2]. Sorghum varieties with longer mesocotyls have been shown to have greater sowing tolerance and higher emergence rates [1]. Therefore, breeding for long mesocotyls is of significant practical importance. In this study, we found that variations in ML were observed in sorghum germplasm (Figure S1), providing a valuable resource for breeding. In order to enhance breeding efficiency through molecular-assisted breeding, it is crucial to identify key genes and their natural variations.

### 3.1. Identification of Candidate Genes Regulating Mesocotyl Length

To date, although over 40 QTLs for mesocotyl length have been reported in rice through QTL mapping and GWAS [11,12,13], related research in sorghum remains limited. However, related research in sorghum remains limited. In this study, 10 significant SNPs associated with mesocotyl length were identified by GWAS among a diverse worldwide collection of 232 sorghum germplasm resources. Among these, two major QTLs with the highest −log10(*p*) values were located on chromosome 4 and chromosome 7, named *qML4-2* and *qML7-1*, respectively (Figure 1A,B). Focusing on *qML7-1*, we found that this region harbors three high-confidence candidate genes (Figure 1C; Table S2). Further screening of three candidate genes on chromosome 7 by transcriptome data revealed that only *LOC8073540* exhibited significant upregulation in sorghum with long mesocotyls compared with those with short ones (Figure 1D, Table S2). Developmental expression analysis further confirmed that the expression of *LOC8073540* was significantly higher in a long-mesocotyl variety and showed an increasing trend during mesocotyl development (Figure 1E). Given that an ERF transcription factor (*LOC_Os09g20350*) has been implicated in mesocotyl elongation in rice [15], we considered that *LOC8073540* (*SbERF60*) is a strong candidate gene for ML regulation.

Light is one of the major external factors regulating mesocotyl elongation. Mesocotyl elongation is significantly suppressed under light conditions but strongly promoted in the dark, and light signaling pathways are known to be involved in regulating hypocotyl/coleoptile elongation in plants. In *Arabidopsis*, the negative regulators of photomorphogenesis, COP1 and the transcription factors PIFs, promote hypocotyl elongation, whereas the positive regulator HY5 suppresses it [24,27]. Additionally, CBF1 promotes hypocotyl elongation by integrating light and cold signals through enhancing the accumulation of PIF4 and PIF5 proteins [24]. In maize, ZmSRO1e promotes mesocotyl elongation by inhibiting the transcriptional activity of ZmbZIP61, a homolog of HY5, on downstream cell elongation-related genes [27]. Moreover, light can coordinate with phytohormones to regulate mesocotyl growth and development [28].

Further characterization showed that SbERF60 features a conserved AP2 domain and is expressed in all seedling organs, with the highest transcript levels in the mesocotyl of etiolated seedlings, indicating that its expression is affected by light. This light-responsive pattern, together with its enrichment in the etiolated mesocotyl, suggests that SbERF60 plays an important role in regulating mesocotyl elongation possibly through a light-signaling-related pathway. Subcellular localization assays indicated that SbERF60 is present in both the nucleus and cytoplasm. Although this dual distribution is unusual for a transcription factor, similar observations have been reported for other plant ERFs [29,30]. Collectively, these findings suggest that SbERF60 plays a key role in regulating mesocotyl length in sorghum.

### 3.2. Natural Variation in the SbERF60 Promoter Is Responsible for SbERF60 Expression Regulation

Modern breeding is built on understanding how natural genetic variation in key genes underlies phenotypic diversity. Therefore, identifying these variants and pinpointing superior alleles is fundamental [31]. To facilitate the application of *SbERF60* in sorghum deep-sowing tolerance breeding, we identified elite haplotypes of this gene. Based on the resequencing data from 232 core sorghum accessions, we identified 35 SNPs in the *SbERF60* promoter region, which formed three major haplotypes (Hap1, Hap2, Hap3) (Figure 3A,B).

Further phenotypic analysis indicated that accessions carrying Hap1 had a significantly shorter mesocotyl length than those with Hap2 or Hap3, and *SbERF60* expression was correspondingly lower in Hap1 accessions (Figure 3B,C). Consistently, luciferase assays demonstrated that the promoter activity of Hap3 was significantly higher than that of Hap1 and Hap2 (Figure 3D). These findings collectively demonstrate that natural sequence variation in the *SbERF60* promoter modulates its transcriptional activity, thereby influencing mesocotyl length.

### 3.3. SbERF60 Promotes Mesocotyl Elongation and Improves Deep Seeding Emergence

The mesocotyl pushes the shoot of the seedling out of the soil during seed germination; its growth is highly related to deep sowing tolerance. Germplasm with longer mesocotyls can enhance sorghum tolerance to deep sowing [1]. Research has demonstrated that ERFs are crucial regulators of hypocotyl/mesocotyl growth in plants. CBF1 promotes hypocotyl elongation by integrating light and temperature signals in *Arabidopsis* [24]. AtERF71 promotes hypocotyl elongation, while ERF72 negatively regulates hypocotyl elongation by interacting with ARF6 and BZR1 [22,23]. In rice, an ERF transcription factor (*LOC_Os09g20350*) was identified as a high-confidence candidate gene regulating mesocotyl elongation through GWAS and QTL mapping [15].

In a previous study, we found that five ERF genes may be key regulators of ML in sorghum [26]. To further clarify the functions of *SbERF60*, we generated transgenic rice lines overexpressing *SbERF60*. These lines exhibited significantly longer mesocotyls than wild-type plants under both 6 cm and 8 cm sowing depths (Figure 4A–D). At a sowing depth of 1 cm, normal mesocotyl growth is enough for seedlings to emerge, so extra growth driven by *SbERF60* provides no clear benefit. At a depth of 6 cm, however, deep sowing creates stress through darkness and soil pressure, which limits emergence in wild-type plants. Under these conditions, SbERF60 overexpression promotes additional mesocotyl elongation, helping seedlings push through the soil. Correspondingly, the overexpression lines also showed a markedly higher emergence rate under deep-sowing conditions (6 cm) (Figure 5A,B). These results demonstrate that *SbERF60* enhances deep-sowing tolerance by promoting mesocotyl elongation in rice.

## 4. Materials and Methods

### 4.1. Plant Materials and Growth Conditions

In this study, a panel of 232 sorghum accessions described in our earlier study was used for GWAS [25]. These accessions were cultivated and harvested in 2021 at a site in Jinzhong, Shanxi Province, China (112°42′ E, 37°36′ N). For the dual luciferase reporter assays, three-week-old *N. benthamiana* plants were cultivated and utilized in the study. These plants were grown at 24 °C under a 16 h light/8 h dark photoperiod. For spatial expression analysis, 5-day-old seedlings were grown at 28 °C under either complete darkness (for etiolated seedlings) or a 16 h light/8 h dark photoperiod (for green seedlings). The SbERF60 overexpressing transgenic lines (*pUbi:SbERF60*) were generated in the Zhonghua 11 background.

### 4.2. Measurement of ML

The mesocotyl length of sorghum accessions was determined as follows. Sorghum seeds were planted in plastic trays containing nutrient soil at a depth of 2 cm. The mesocotyl length of seedlings was assessed using a ruler following careful extraction and water rinsing at the 8-day post-sowing stage. For each of the three biological replicates, a minimum of six seedlings were assessed. The mesocotyl length of rice was determined as follows. The seeds of wild-type ZH11 and transgenic lines were planted in plastic containers containing a blended substrate composed of equal parts nutrient-rich soil and silica sand at planting depths of 6 cm and 8 cm. The length of the mesocotyl was determined for each seedling with a ruler 10 days following sowing. Seedlings of sorghum and rice used for ML measurement were maintained in complete darkness at a constant temperature of 28 °C. Statistical analyses were performed using R v.4.3.0 (R Foundation for Statistical Computing, Vienna, Austria).

### 4.3. GWAS Analysis

GWAS has provided details of the genotype data, phylogenetic tree, LD decay, principal component analysis, and population structure of the 232 sorghum accessions [25]. Association analysis between molecular markers and mesocotyl length was performed using EMMAX (beta-07Mar2010) under a mixed linear model (LMM), with results displayed via Manhattan and Q-Q plots.

Given that the false discovery rate–adjusted *p* value of 0.05 was excessively restrictive, an unadjusted *p* < 1 × 10^−6^ was used as a less stringent threshold to declare marker-trait associations (MTAs). At each significant locus, the SNP exhibiting the minimum *p* value was designated as the candidate SNP. Then candidate genes were identified as those located near significant SNP loci and were functionally annotated based on the National Center for Biotechnology Information (NCBI database) (https://ftp.ncbi.nlm.nih.gov/genomes/all/GCF/000/003/195/GCF_000003195.3_Sorghum_bicolor_NCBIv3, accessed on 15 January 2023). Furthermore, genome-wide linkage disequilibrium was independently computed with TASSEL 3.0 [32].

### 4.4. Transcriptome Data Analysis

ICSV219, TCSV361, Yidumi, and Jinhui75 were used for RNA-seq analysis [26]. RNA extraction, library construction, sequencing, and sequence analysis were performed as previously described [26]. The reference genome and gene annotation files were downloaded from the genome website (https://ftp.ncbi.nlm.nih.gov/genomes/all/GCF/000/003/195/GCF_000003195.3_Sorghum_bicolor_NCBIv3, accessed on 20 December 2022). Gene expression levels were normalized as fragments per kilobase per million reads (FPKM). Clustering analysis of differentially expressed genes among samples was conducted using the R package pheatmap (version 1.0.8) [26].

### 4.5. DNA Constructs and Plant Transformation

The complete coding region of *SbERF60* (without the stop codon) was PCR-amplified employing KOD polymerase and subsequently cloned into the *pBWA(V)HU* plasmid, harboring the ubiquitin promoter and a Flag tag sequence (Toyobo, Osaka, Japan). The genetic transformation to produce *SbERF60* overexpressing transgenic lines was performed by Wuhan Biorun BioSciences, Wuhan, Hubei, China. The *SbERF60* overexpressing transgenic lines were identified by amplifying the hygromycin resistance gene from DNA. Details of the vector construction and primers used for transgene detection are provided in Table S1.

### 4.6. Deep Seedling Emergence Assessment

For deep-seeding simulation with standardized soil conditions, a 1:1 (*v*/*v*) mixture of nutrient soil and silica sand was used as the growth substrate. From each line, ten healthy and plump seeds were selected and sown at depths of 1 cm and 6 cm. Emergence of seedlings was recorded each day. Statistical significance between groups via one-way ANOVA with Tukey’s HSD post hoc test (*p* < 0.05). All data are presented as mean ± SD, with *n* = 3 biological replicates per haplotype and at least 6 seedlings per replicate.

### 4.7. Subcellular Localization of SbERF60

The complete coding region of SbERF60 (excluding the stop codon) was inserted into the *pBWA(V)HU* vector to create a GFP-tagged expression construct. Subsequently, the recombinant vector was introduced into *N. benthamiana* protoplasts following a previously described protocol. The *pBWA(V)HU* empty vector was transformed into the *N. benthamiana* protoplasts and used as a negative control. After 18 h of dark incubation at 28 °C, protoplasts were observed with a confocal laser scanning microscope. The primers used are listed in Table S1.

### 4.8. Haplotype Analysis

A list of SNPs in the promoter region of SbERF60 (2500 bp upstream) was extracted from the genotype data. LD analysis of these SNP loci was conducted using HaploView software (version 4.2), with an r^2^ > 0.8 set as the threshold for tight linkage. Tag SNPs significantly associated with sorghum mesocotyl length were thereby identified. The phenotypic differences in mesocotyl length corresponding to the different alleles of these Tag SNPs were then evaluated for significance using the Student’s *t*-test in GraphPad Prism v8.0. SNPs exhibiting significant allelic effects were subsequently considered candidate functional SNPs. Differences in mesocotyl length among haplotypes formed by these candidate functional SNPs were further examined via one-way ANOVA with Tukey’s HSD post hoc test (*p* < 0.05). All data are presented as mean ± SD, with *n* = 3 biological replicates per haplotype and at least 6 seedlings per replicate.

### 4.9. RT-PCR

Total RNA was isolated using the GREENspin Plant RNA Extraction kit (ZP432, Beijing, China). A reverse transcriptase kit was employed to synthesize first-strand cDNA starting with 2 μg of total RNA. Quantitative real-time PCR was subsequently performed on a Light Cycler 96p (Kunpeng, Singapore) using SYBR Premix Ex Taq (TaKaRa, Shiga, Japan). In the assay, *SbEIF4A* served as the internal reference gene. The experiment included three biological replicates, all showing similar results. Primers used for qRT-PCR are provided in Table S1.

### 4.10. Cloning of SbERF60 Promoter and Dual Luciferase Reporter Assay

Three distinct haplotypes of the SbERF60 promoter (2500 bp) were cloned and individually inserted into the pGreenII 0800-LUC vector. After transformation into *Agrobacterium tumefaciens GV3101*, each construct was used to perform infiltration assays in *N. benthamiana* leaves. To assess transcriptional activity, we measured firefly luciferase (LUC) and Renilla luciferase (REN) activities using the Dual-Luciferase Reporter Assay System (Vazyme, DL101-01, Nanjing, China) and captured luminescence images using a plant imaging system (NightSHADE LB985, Berthold Technologies, Bad Wildbad, Germany).

## 5. Conclusions

This study identified SbERF60 as a key regulator of mesocotyl elongation in sorghum. We demonstrated that natural variation in its promoter, particularly the superior Hap3 haplotype, enhances SbERF60 expression by increasing promoter activity, leading to longer mesocotyls. Functional validation confirmed that *SbERF60* promotes mesocotyl elongation and significantly improves deep-seeding emergence in rice. These findings provide both a genetic resource and a promising target for molecular breeding aimed at enhancing seedling emergence in sorghum.

## Data Availability

The original contributions presented in this study are included in the article/Supplementary Material. Further inquiries can be directed to the corresponding author.

## Supplementary Materials

The following supporting information can be downloaded at: https://www.mdpi.com/article/10.3390/plants15132000/s1. Figure S1: Frequency distribution plot of mesocotyl length used for GWAS across 232 sorghum accessions; Figure S2: Phenotypic variation in mesocotyl length among diverse sorghum varieties. Arrows indicate the coleoptilar nodes; Figure S3: Expression difference of the candidate genes for mesocotyl length by RNA-seq; Figure S4: DNA identification results of the SbERF60 overexpressing transgenic lines; Table S1: RT-qPCR and vector construction primers used in the study; Table S2: Candidate genes on chromosome 7 region detected by GWAS; Table S3: The expression level of *SbERF60* in 3 HAPs.
