# Supplementary figures and images for: Genome-Wide Association Uncovered SbERF60 Positively Regulates Mesocotyl Length in Sorghum
Source: Plants (Basel). 2026 Jun 28;15(13):2000. doi: 10.3390/plants15132000 (PMC13363829; doi:10.3390/plants15132000)

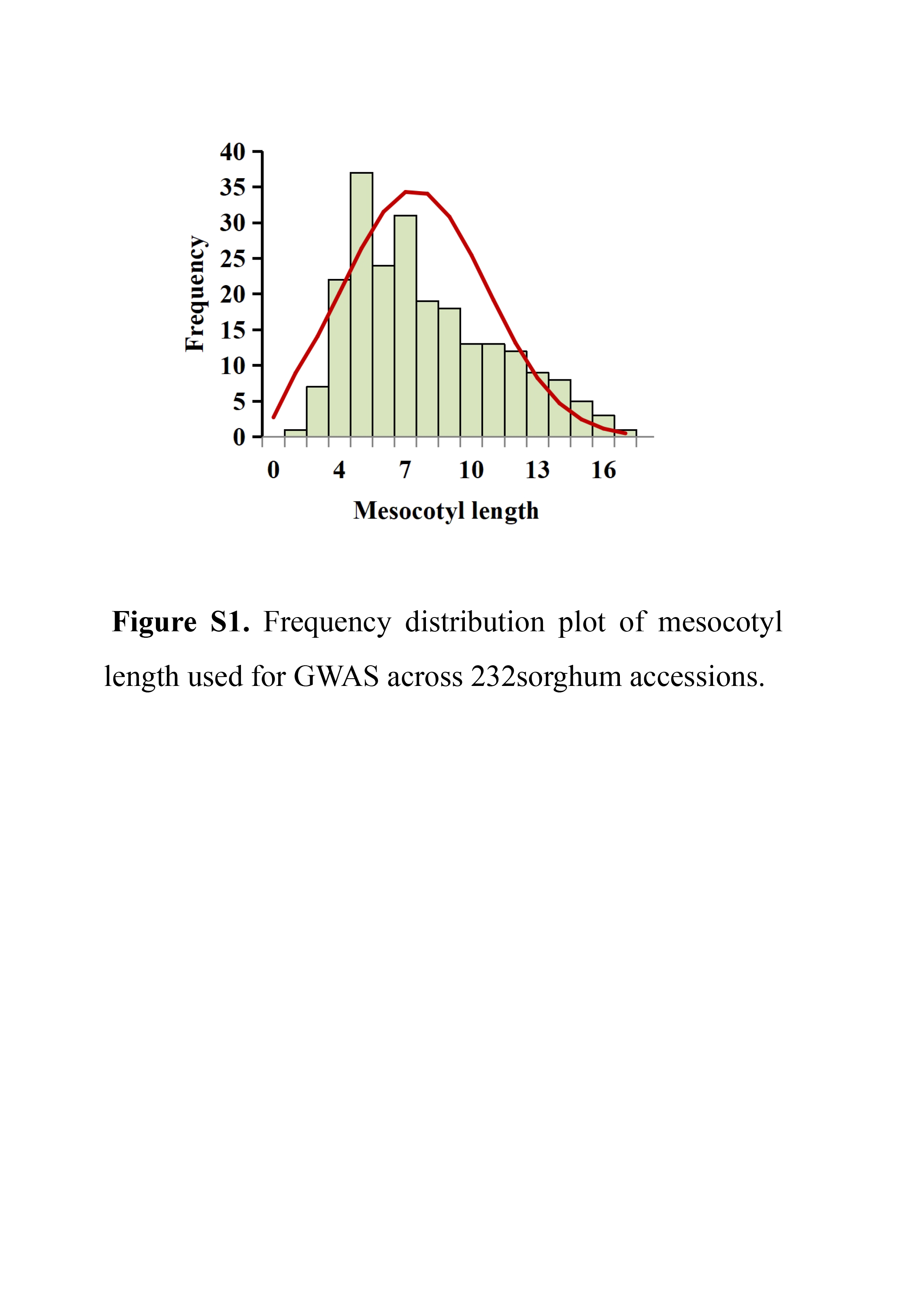

Supplement: Supplementary file 1 [file plants-15-02000-s001.zip › Supplemental figures-S1.tif]

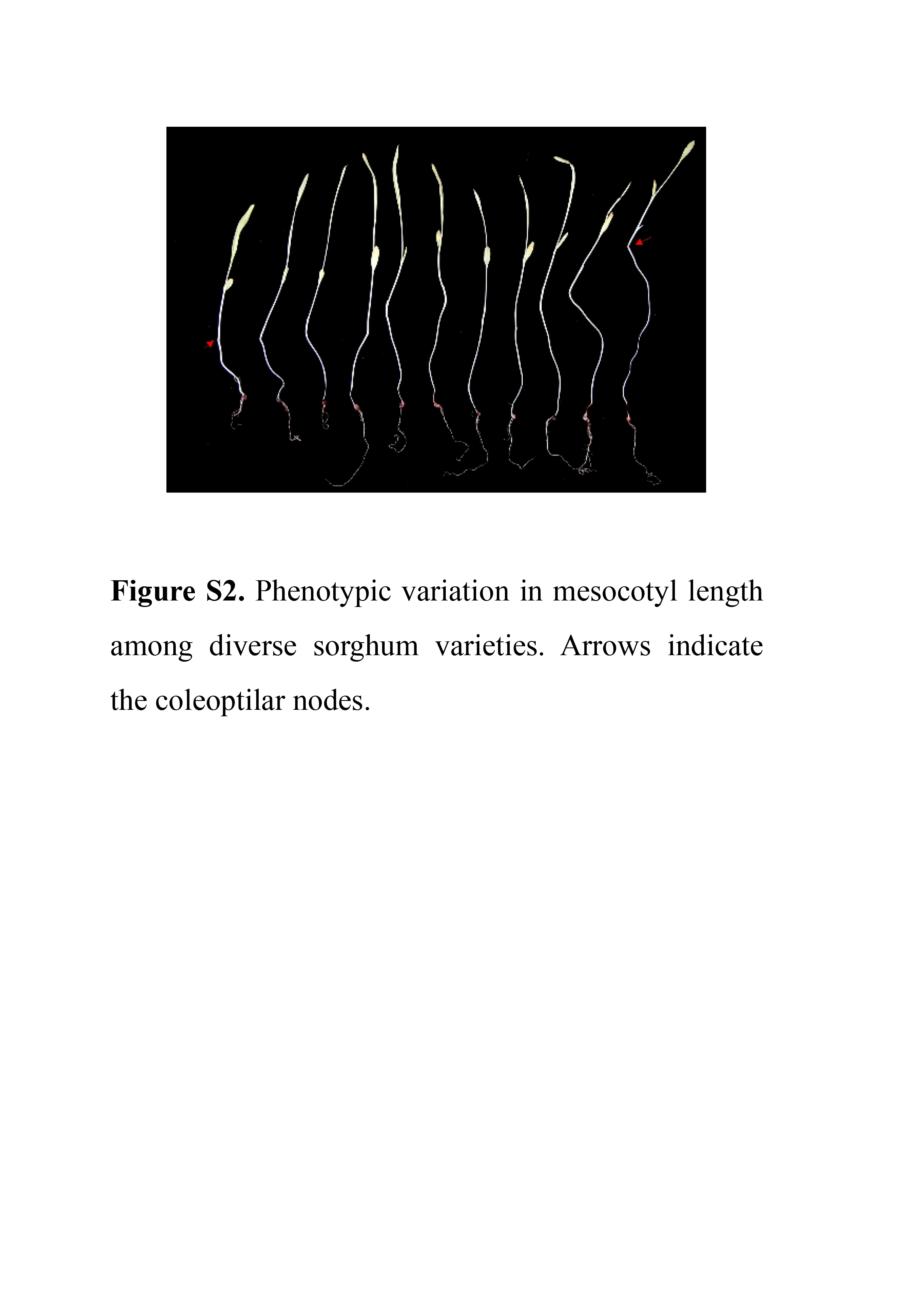

Supplement: Supplementary file 1 [file plants-15-02000-s001.zip › Supplemental figures-S2.tif]

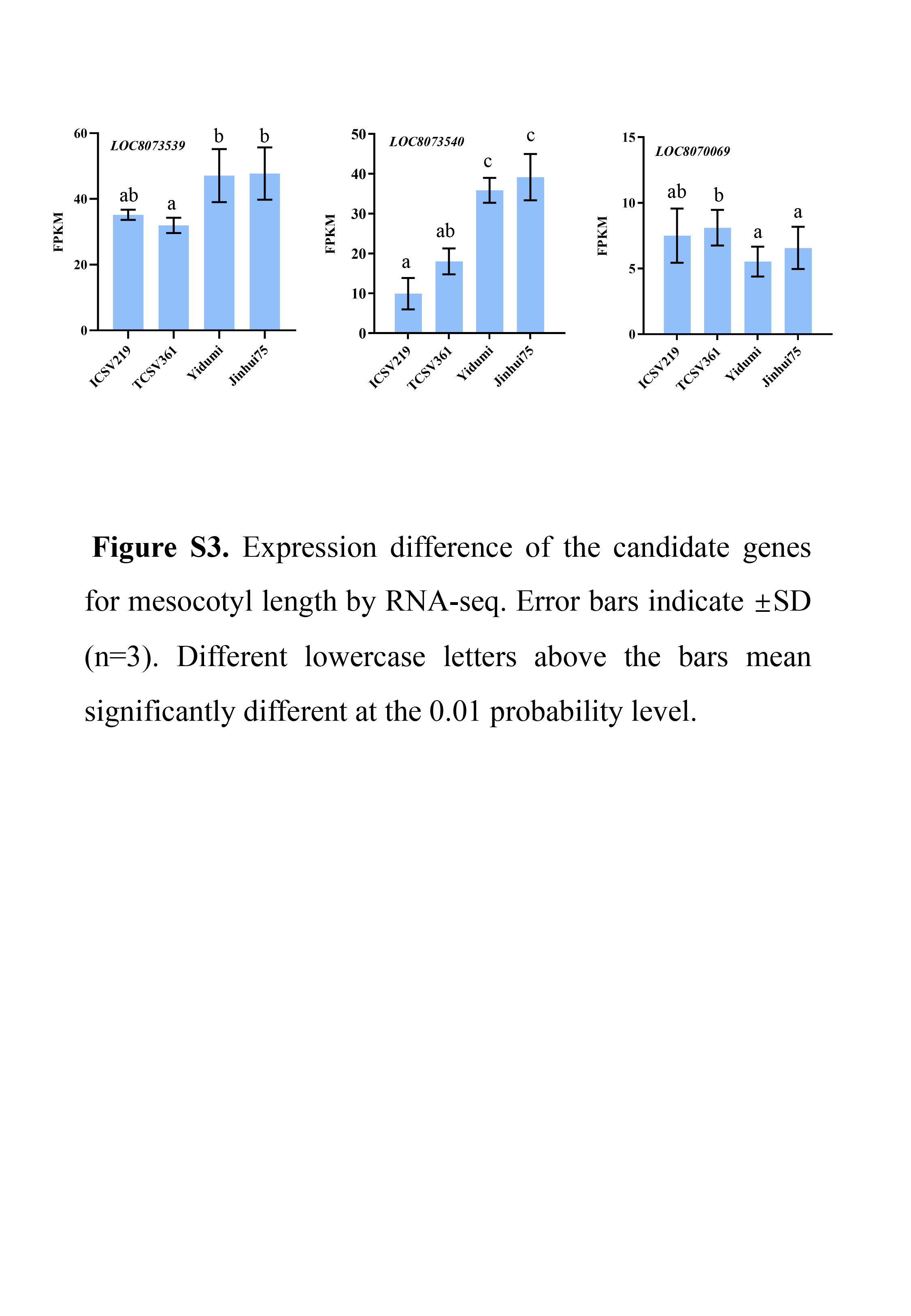

Supplement: Supplementary file 1 [file plants-15-02000-s001.zip › Supplemental figures-S3.tif]

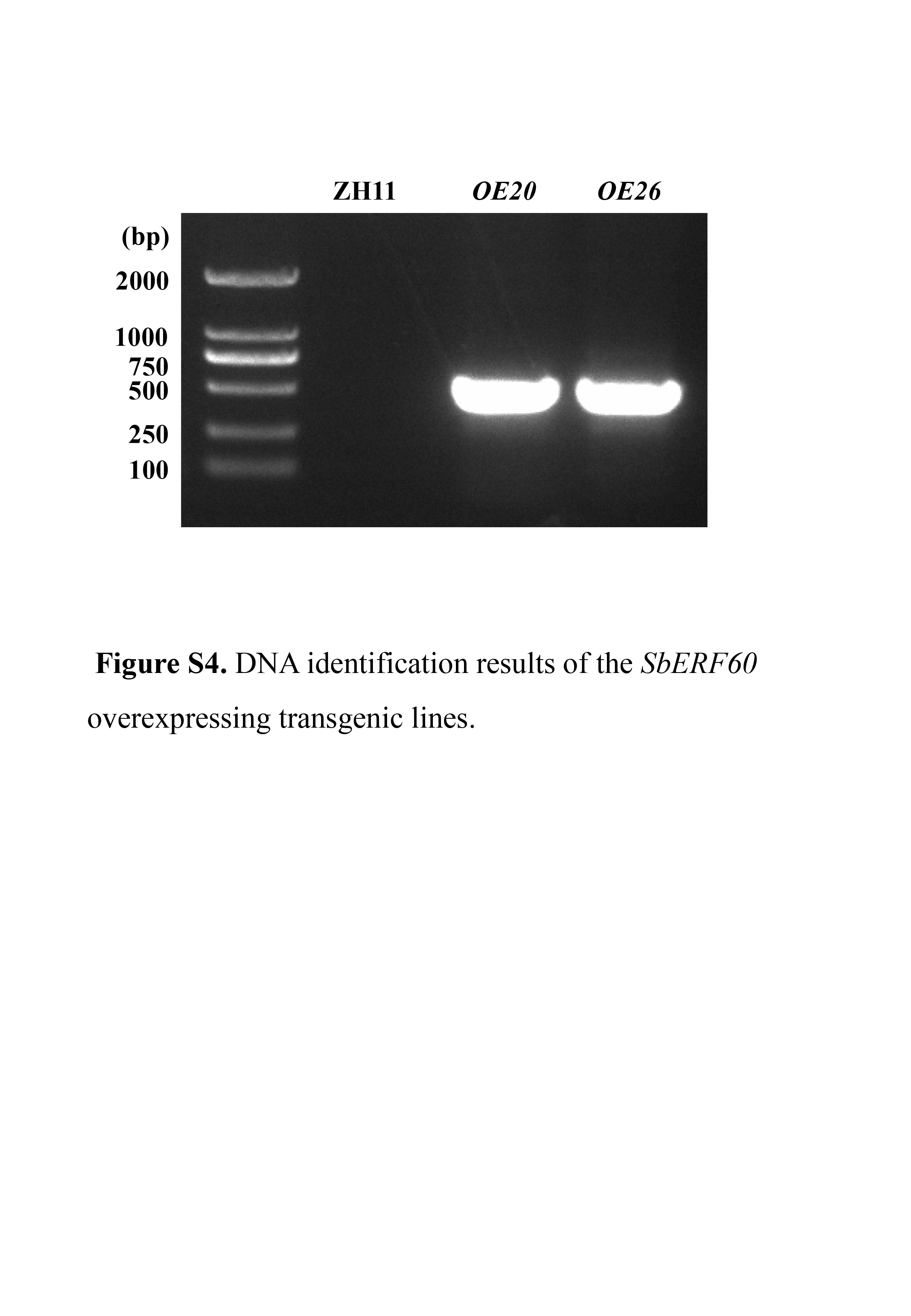

Supplement: Supplementary file 1 [file plants-15-02000-s001.zip › Supplemental figures-S4.tif]
